# The Sarracenia Purpurea, a Remedy for Small-Pox

**Published:** 1862-07

**Authors:** Frederick W. Morris

**Affiliations:** Resident Physician of the Halifax Visiting Dispensary


### THE SARRACENIA PURPUREA, A REMEDY FOR SMALL-POX.

To the Editor of the American Medical Times:—

Sir:—You have by this time, in all probability, heard something of an extraordinary discovery for the cure of small-pox, by the use of "Sarracenia Purpurea," or Indian Cup, a native plant of Nova Scotia. I would beg of you, however, to give full publicity to the astonishing fact, that this same humble bog-plant of Nova Scotia is the remedy for small-pox, in all its forms, in twelve hours after the patient has taken the medicine. It is also as curious as it is wonderful that, however alarming and numerous the eruptions, or confluent and frightful they may be, the peculiar action of the medicine is such that very seldom is a scar left to tell the story of the disease.

I will not enter upon a physiological analysis now; it will be sufficient for my present purpose to state, that it cures the disease as no other medicine does—not by stimulating functional re-agency, but by actual contact with the virus in the blood, rendering it inert and harmless, and this I gather from the fact that if either vaccine or variolous matter be washed with the infusion of the Sarracenia, they are deprived of their contagious properties. The medicine, at the same time, is so mild to the taste that it may be mixed largely with tea or coffee, as I have done, and given to connoisseurs in these beverages to drink, without their being aware of the admixture.

Strange, however, to say, it is scarcely two years since science and the medical world were utterly ignorant of this great boon of Providence; and it would be dishonorable in me not to acknowledge that had it not been for the discretion of Mr. John Thomas Lane, of Lanespark, County Tipperary, Ireland, late of Her Majesty's Imperial Customs of Nova Scotia, to whom the MecMac Indians had given the plant, the world would not now be in possession of the secret. No medical man before me had ever put this medicine upon trial; but in 1861, when the whole Province of Nova Scotia was in a state of panic, and patients were dying in the hospitals at the rate of twelve and a-half per cent., from May to August, Mr. Lane, in the month of May, placed the "Sarracenia" in my hands to decide upon its merits; and after my trials then and since, I have been convinced of its astonishing efficacy.

The Indian Cup is found in swamps and moss bogs. Its capacious globular receptacles are generally filled with cool, bland water. The Cups are lined with bristles pointing downwards, that entangle the flies that come to drink, so that few escape drowning. It is a very curious and remarkable family of plants exclusively North American, and not to be met with west of the Alleghanies. The leaves take the form of a long bulbous tube or funnel, like the bowl of a tobacco-pipe, terminating with a hood-shaped appendage not unlike an Indian squaw's cap. The flowers, with their hard involuted crenate calyx, and fine sessile segments, like the yellow water-lily, deep crimson stigmata, and corresponding stamina, in form and appearance are very remarkable. All of the tribe inhabit marshy grounds. The "Sarracenia Purpurea" is the most common species, and like all the beautiful things of Providence, widely diffused from Hudson's Bay to the State of North Carolina.* The root consists of numerous short radicles, fibrous and stringy, which when powdered, have a very faint and agreeable aroma, with a taste very like the willow alkaloid, or salicin. The dose of the medicine —the powdered root—is about a dessert-spoonful, simmered in a pint of water down to half a pint; this is divided into two doses, one taken immediately, the other in six hours; no sugar should be given with it. The only functional influence it seems to have, is in promoting the flow of urine, which soon becomes limpid and abundant, and this is owing perhaps to the defecated poison or changed virus of the disease exclusively escaping

*[The Sarracenia Purpurea grows in the swampy lands of New Jersey, and is called the "Pitcher plant," and "Side-saddle flower;" Griffith does not mention it in his "Medical Botany." Various southern species exist, and two of these are noticed in the U.S. Dispensatory.—Ed. Am. Jour. Pharm.]

through that channel. The "Sarracenia," I take reason to believe a powerful antidote for all contagious diseases, lepra, measles, varicella, plague, contagious typhus, and even syphilis, also a remedy in jaundice. I am strongly inclined to think it will one day play an important part in all these. Yours, etc.,

FREDERICK W. MORRIS, M.D.,
Resident Physician of the Halifax Visiting Dispensary.

—*Am. Med. Times, May* 24, 1862.

